# Artificial Intelligence and Big Data Technologies in the Construction of Surgical Risk Prediction Model for Patients with Coronary Artery Bypass Grafting

**DOI:** 10.1155/2023/9575553

**Published:** 2023-07-07

**Authors:** Xiaoqiang Tang, Tao Wang, Haifeng Shi, Ming Zhang, RuoHan Yin, Qiyong Wu, Changjie Pan

**Affiliations:** ^1^Radiology Department, the Affiliated Changzhou No. 2 People's Hospital of Nanjing Medical University, Changzhou 213164, Jiangsu, China; ^2^Cardio Thoracic Department, the Affiliated Changzhou No. 2 People's Hospital of Nanjing Medical University, Changzhou 213164, Jiangsu, China

## Abstract

The objective of this work was to predict the risk of mortality rate in patients with coronary artery bypass grafting (CABG) based on the risk prediction model of CABG using artificial intelligence (AI) and big data technologies. The clinical data of 2,364 patients undergoing CABG in our hospital from January 2019 to August 2021 were collected in this work. Based on AI and big data technology, business requirement analysis, system requirement analysis, complication prediction module, big data mining technology, and model building are carried out, respectively; the successful CABG risk prediction system includes case feature analysis service, risk warning service, and case retrieval service. The commonly used precision, recall, and F1-score were adopted to evaluate the quality of the gradient-boosted tree (GBT) model. The analysis proved that the GBT model was the best in terms of precision, F1-score, and area under the receiver operating characteristic curve (ROC). According to the CABG risk prediction model, 1,382 patients had a score of <0, 463 patients had a score of 0 ≤ score ≤ 2, 252 patients had a score of 2 < score ≤ 5, and 267 patients had a score of >5, which were stratified into four groups: A, B, C, and D. The actual number of in-hospital deaths was 25, and the in-hospital mortality rate was 1.05%. The mortality rate predicted by the CABG risk prediction model was 2.67 ± 1.82% (95% confidential interval (CI) (2.87–2.98)), which was higher than the actual value. The CABG risk prediction model showed the credible results only in group B with AUC = 0.763 > 0.7. In group B, 3 patients actually died, the actual mortality rate was 0.33%, and the predicted mortality rate was 0.96 ± 0.78 (95% CI (0.82–0.87)), which overestimated the mortality rate of patients in group B. It successfully constructed a CABG risk prediction model based on the AI and big data technologies, which would overestimate the mortality of patients with intermediate risk, and it is suitable for different types of heart diseases through continuous research and development and innovation, and provides clinical guidance value.

## 1. Introduction

Coronary artery bypass grafting (CABG) is one of the surgical methods commonly used in the clinical treatment of coronary heart disease (CHD). It can significantly relieve the symptoms of chest tightness, shortness of breath, and exercise limitation caused by coronary ischemia in patients with CHD, and reduce the incidence of angina pectoris and myocardial infarction [1–3]. As the number of patients undergoing CABG increases year by year, some patients may even need to undergo bypass surgery to relieve their condition, which makes cardiac surgeons face severe challenges in terms of patient condition assessment and surgical risk prediction [4–6]. If the patient's surgical risk can be evaluated in advance, and an individualized treatment plan tailored to the patient's own can be prepared, the success rate of CABG will be greatly improved and the patient's prognosis will be improved. Therefore, establishing a disease risk prediction model based on artificial intelligence and big data analysis technology is of high meaning [7, 8]. Preoperative risk prediction is a method to quantitatively evaluate the risk level according to the physiological status of patients, basic diseases, the effects of anesthesia, and surgery on the body. According to the risk level of patients, anesthesiologists formulate and revise anesthesia and perioperative medical plans and take preventive measures, so as to reduce potential risks and improve safety and medical care quality.

With the development and wide application of electronic technology, a large-scale information database integrating multicenter has been established and improved [9–11]. Scholars have made a statistical analysis of large sample data from multiple centers so that the related research on CABG risk assessment has gradually developed from a single-center study to multicenter joint research, and the research scale has expanded from the number of thousands of cases to hundreds of thousands. At present, there are more than ten kinds of cardiac surgery risk scoring systems, among which EuroSCORE, Cleveland risk-scoring system, and Ontario risk-scoring (OPR) system are commonly used internationally [12–14]. Compared with patients in Western developed countries, Chinese patients are different in demographics, comorbidities, preoperative risk factors, disease course, and even medication compliance. Moreover, compared with Western bypass grafting, most of the bypass grafting in China is off-pump. Therefore, the cardiac surgery risk assessment system based on patient data in Western countries cannot better assess the surgical risk of Chinese patients [15–18]. Therefore, it is urgent for doctors to find a breakthrough in the surgical risk prediction mode with the help of machine learning, big data analysis, and other technologies.

Therefore, this work collected the clinical data of patients undergoing CABG in the Affiliated Changzhou No.2 People's Hospital of Nanjing Medical University from January 2019 to August 2021, conducted business demand analysis, system demand analysis, complication prediction module, big data mining technology, and model establishment, and successfully constructed the risk prediction model of CABG based on AI and big data technology, so as to test the application performance of this model for patients undergoing CABG in our center, and provide a reference for clinical selection of risk prediction model of CABG.

## 2. Materials and Methods

### 2.1. Research Objects

In this work, it collected the clinical data of 2,364 routine CABG patients in the Affiliated Changzhou No.2 People's Hospital of Nanjing Medical University from January 2019 to August 2021. It mainly included the basic demographic data, preoperative medical history, preoperative laboratory examination data, coronary angiography data, cardiac ultrasound data, surgical data, postoperative complications, and discharge follow-up data. Death was the primary study endpoint. The CABG risk prediction model based on AI and big data technologies constructed in this work was applied to calculate the score of each patient.

### 2.2. Business Need Analysis

**Figure 1:**
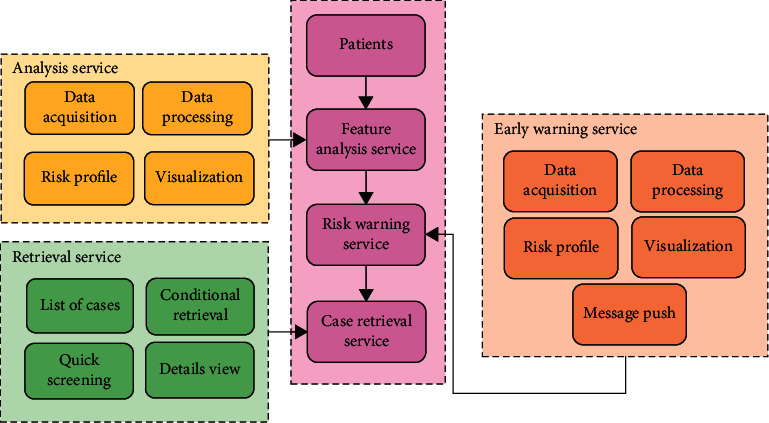
Service process of CABG risk prediction model.

The CABG risk prediction model needed to implement three parts of services, namely, case feature analysis service, risk early-warning service, and case retrieval service. The service process of the CABG risk prediction model is shown in Figure 1.

The model included an efficient and intuitive case feature analysis service that assisted clinicians in assessing the risk of complications in a case. First, it was necessary to use the data acquisition module to automatically collect the relevant data of the cases, adopt the data processing module to filter and clean the characteristic data of the cases, and then apply the feature engineering of the myocardial infarction prediction module to mine and analyze the key factors. Finally, the output results were displayed with the visualization module. Clinicians can evaluate the distribution of key pathogenic factors and risk factors based on the visualization module, such as the level of factor scores and the degree of fit of factor values.

The model included a risk early warning service, which can assist clinicians in monitoring the risk of complications in cases. The service needed to go through the data acquisition module and data processing module step by step, adopt the risk prediction module to realize the binary classification of the occurrence of complications, and finally display the output results in the form of probability. Different colors represented different probability results. The larger the probability value, the darker the color. If the probability value exceeded the configured threshold, an alert message would be pushed. The message push module can be configured according to the administrator's rules, and send multichannel messages such as phone calls, text messages, and emails.

The case retrieval service needed to complete the rapid screening of in-hospital case data and retrieval of combined conditions, provide case diagnosis and treatment data and medical record details, assist doctors in quickly locating cases, and finally select the most appropriate diagnosis and treatment methods and management plans based on the analysis and prediction results and medical record records.

### 2.3. System Requirement Analysis

The users of the model may be algorithm engineers, scientific research analysts, system administrators, medical staff, log reviewers, etc.

The system database needed to have both massive data retrieval and business data storage capabilities at the same time. The data table structure had 4 parts, including basic data, case information, management information, and log security. The basic data included users of different roles and basic data dictionaries. Case information included case visit information, medical history, and the diagnosis and treatment data. Management information included task configuration, permission configuration, and scheduling policy data. Log security included system operation, operation, and data processing.

The overall deployment of the system was carried out in the intranet environment; the platform was connected with the large dataset of the hospital; and the integrated platform service gateway was adopted to complete the external network access and message push, so as to realize the security authentication management using the unified portal.

### 2.4. Overall System Design

**Figure 2:**
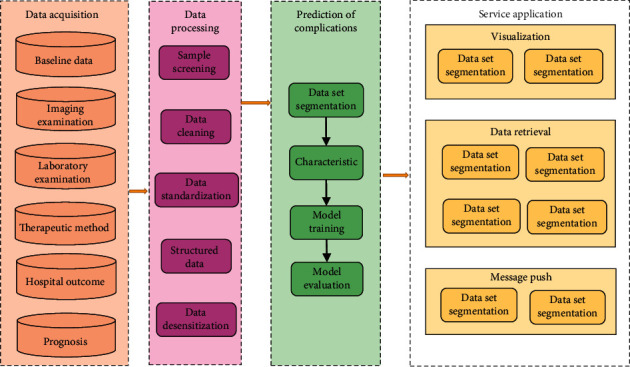
Overall architecture diagram of the model.

The model constructed in this work adopted a browser/server (B/S) architecture. The front-end view, business application logic, and back-end data are separated using SpringBoot layered technology. The overall system architecture is shown in Figure 2. The Vue + ElementUI technology was used to achieve full compatibility of front-end browsers with different types of browsers such as IE, Chrome, and Firefox. Using the database access object technology, according to different business and scenarios, the back-end server uses the underlying basic units to stitch together business logic, including modules such as data collection and processing, complication prediction, visualization, and message push. The database is composed of MySQL and Elasticsearch. The MySQL stored business data, which was highly feasible, and the Elasticsearch, as a storage layer for massive clinical diagnosis and treatment data of cases [19], used a high-performance distributed architecture to improve data query efficiency.

### 2.5. Complication Prediction Module

**Figure 3:**
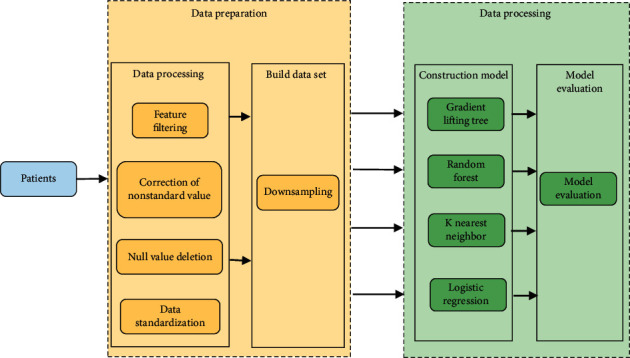
The route diagram for building a complication prediction model.

The complication prediction model was established by using the supervised machine learning algorithms. Medical big data was complex and specific, and most of it was an unbalanced dataset. This dataset contained 2364 positive cases and 5387 negative cases. Therefore, when a model was trained, the optimization of the model often lied in the change of the algorithm, the iteration of the hyperparameters, and especially the construction of the dataset that needs to be incorporated into the model optimization as a key adjustment method. First, 2,364 cases were randomly sampled from the negative data by the downsampling method to maintain the balance with the positive data and avoid learning bias. Then, the data were divided into a training set and a test set in a ratio of 6 : 4 on the dataset. Finally, multiple models were constructed with random forest (RF), GBT, logistic regression (LR), and K-nearest neighbor (KNN) algorithms. The F1-score was undertaken as the evaluation index to select the optimal solution in each model as the final model. The F1-score was one of the indicators used in machine learning to measure the accuracy of the binary classification model. Compared with the accuracy rate, it had both the precision rate and the recall rate of the classification model, and the real prediction ability of the model was more accurate and objective. The route diagram for building a complication prediction model is shown in Figure 3.

### 2.6. Big Data Mining Technology and Model Building

Big data mining technology is to discover the logical relationship between dependent variables and outcome variables through various classification models. The main steps are data preparation, data cleaning, data mining, result expression, and analysis. Five data mining methods include the regression model, classification model, association model, clustering model, deviation model, and so on. The project team used five data mining methods for analysis, such as rotating random forest, random forest, Bayesian network, and naive Bayesian network support-vector machine.

### 2.7. Model Training for Incremental Learning GBT

The incremental learning GBT batch processing method was adopted to predict the target value of the preset tree structure, and obtain the classification label or predicted value. In this work, (·) represents the loss function of the model. The calculation equation of the loss function of the incremental learning GBT model was as follows:

(1) $$\begin{array}{c} l\left(b,G\left(a\right)\right)=\frac{1}{2}{\left(\sum_{t=0}^{t=\left|T\right|}g^{t}\left(a\right)-b\right)}^{2}. \end{array}$$

In the above equation (1), *G*(*a*)=∑_*t*=0_^*t*=|*T*|^*g*^*t*^(*a*) and *t* represent the *t*-th tree. Being similar to the neural network where only one layer of the network was updated at a time, the incremental learning GBT only modified the weights of the leaf nodes of one tree at a time in the process of backward propagation. Therefore, it had to calculate the minimum value of the loss function of the *t*-th tree with the following equation below:

(2) $$\begin{array}{c} l_{\min}^{t}=l\left(b^{t},G_{T/t}\left(a\right)+g^{t}\text{(}\left(x\right)\right). \end{array}$$

In the above equation (2), *T*/*t* represents all decision trees except the t-th tree in the model. Since the model had to minimize *l*_*min*_^*t*^ by updating the *g*^*t*^(*x*), the partial derivative of *l*_*min*_^*t*^ had to be calculated during the training. If *f*=*∂l*^*t*^/*∂g*^*t*^(*x*), *f* represents the first derivative of the loss function, and the equation for the model to calculate the new t-th tree was as follows:

(3) $$\begin{array}{c} {\bar{g}}^{t}\left(x\right)=g^{t}\left(x\right)-\delta f. \end{array}$$

In the above equation, *δ* represents the learning rate of the model. When the loss function was the root mean square error, the first derivative *f* of the loss function belonging to the t-th tree can be derived as the following equation:

(4) $$\begin{array}{c} f=\frac{\partial l\left(a^{t},G^{t}\left(a\right)\right)}{\partial g^{t}\left(a\right)}=\left(g^{t}\left(a\right)-b^{t}\right). \end{array}$$

In the above equation (4), *b*^*t*^ was the ideal cumulative value of the remaining trees except for the *t*-th tree. According to the calculation result of the first derivative, it can be generalized to other derivable loss functions.

### 2.8. Evaluation System Application Interface

**Figure 4:**
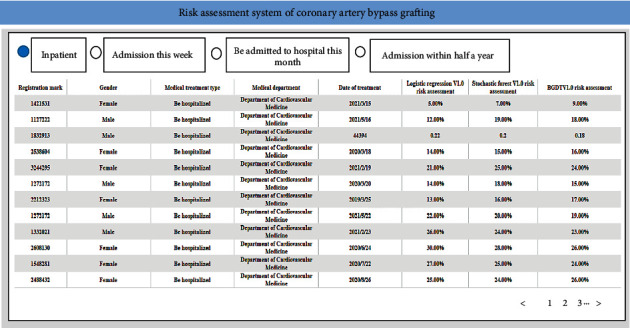
The case patient management interface.

The data acquisition module of the system was connected with the big data integration platform of the hospital. The data acquisition module collected the business data of risk factors in real time, and then used the data processing module to finally summarize and save the data to the complication database [20–22]. The patient management interface includes name, gender, treatment type, treatment department, treatment date, and risk assessment results, as shown in Figure 4.

**Figure 5:**
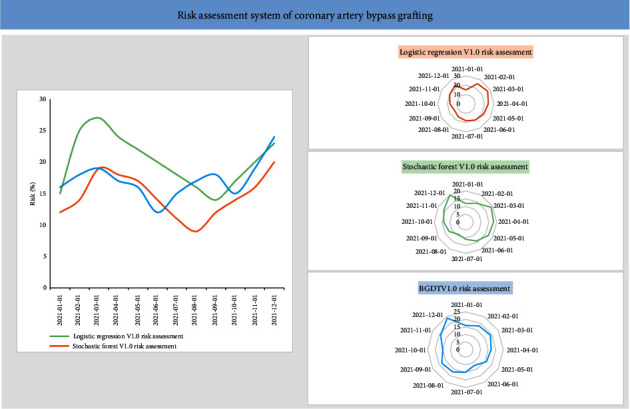
The prediction model management interface.

The model provided interactive access in the form of web pages, providing data query, visual data analysis, and configuration services for scientific researchers, and offering system maintenance, authority allocation, and real-time monitoring functions for system administrators. Based on the clinical data of patients, machine learning algorithms were employed to predict the occurrence of complications, providing doctors with useful information and assisting them in formulating treatment plans for patients. The model management interface includes risk prediction results and risk prediction trends of different models, as shown in Figure 5.

### 2.9. Grouping Methods

The CABG risk prediction model was used to score the patients, and the total score was stratified for cardiovascular risk by the quartile method, and each risk stratification was matched to the corresponding group. The mortality rate of all patients and each group was predicted using the CABG risk prediction model.

### 2.10. Predictive Efficiency Evaluation Methods

The performance of the CABG risk prediction model was analyzed using discrimination and calibration. Discrimination referred to the ability of the model to analyze in-hospital deaths or postoperative survival. Model discrimination was expressed as the area under the receiver operating characteristic curve (AUC). The AUC floated in the range of 0.5–1.0. When AUC ≥0.8, it indicated high reliability, and AUC = 0.7∼0.8 indicated credibility. The H-L goodness of fit was applied to test the calibration power of the model, measuring expected and actual results. When the H-L *P* value >0.05, it indicated that the model had good calibration power. The observed mortality rate was compared with the actual mortality rate, and the observed mortality rate was constructed, which referred to the calibration point of the actual mortality rate ratio [23, 24].

The commonly used precision, recall, and F1-score metrics were selected to evaluate the quality of the GBT model. The specific calculation equations were as follows:

(5) $$\begin{array}{c} \text{Precision}=\frac{{\text{TP}}_{i}}{{\text{TP}}_{i}+{\text{FP}}_{i}},\\ \text{Recall}=\frac{{\mathrm{TP}}_{i}}{{\text{TP}}_{i}+{\text{FN}}_{i}},\\ F1-\text{score}=2\frac{\text{precision}+\text{recall}}{\text{precision}+\text{recall}}. \end{array}$$

In the above equations, *TP*_*i*_ denotes the number of samples that belonged to category *i* and was classified as category *i* by the classifier, *FP*_*i*_ denotes the number of samples that was actually noncategory *i* but was classified into category *i* by the classifier, and *FN*_*i*_ refers to the number of samples that belonged to category *i* but was classified as noncategory *i* by the classifier.

### 2.11. Statistical Methods

All data were expressed in the form of mean ± standard deviation ($\bar{x}$ ± *s*). SPSS 19.0 (IBM Company, America) was used for data analysis. Statistical analysis of basic data was expressed by the chi-square test. *P* < 0.05 means that the difference was statistically significant.

## 3. Results and Discussions

### 3.1. System Model Test Results

**Figure 6:**
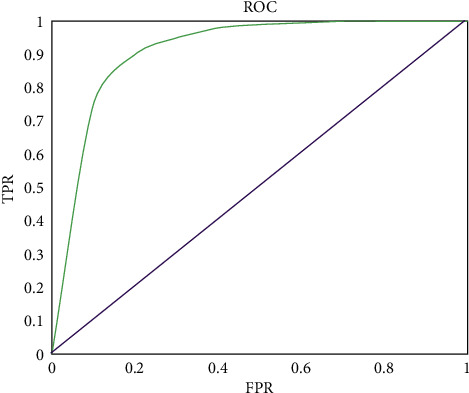
ROC curve of GBT downsampling. Note: the horizontal axis showed a false-positive rate (FPR), and the vertical axis showed a true-positive rate (TPR).

The test results of GBT, RF, LR, and KNN are shown in Table 1. It can be found that compared with the other three models, the GBT was the best model in terms of precision, F1-score, and AUC. The GBT confusion matrix evaluation reflected the stability of the model in all aspects, as shown in Table 2. The ROC curve reflected the strong generalization ability of the system model, as shown in Figure 6.

### 3.2. Analysis Results of the Convergence of the GBT Model

**Figure 7:**
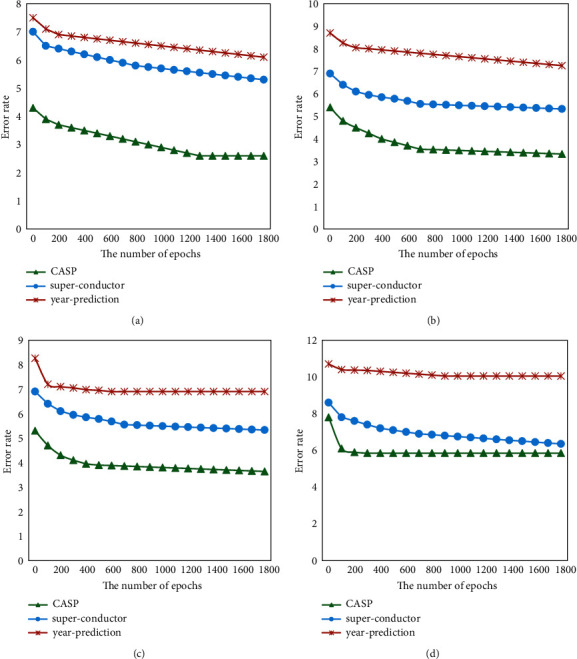
Results of the training error as a function of the number of iterations. (a) Random initialization. (b) Forgetting feature initialization. (c) Median initialization. (d) Information gain-based initialization.

To further verify the convergence of the GBT model, the comparative experiments were performed on three datasets of CASP, superconductor, and year prediction. The depth of the tree was 10, the number of trees was 100, and the number of model iteration rounds was 2000. These three datasets and different tree structure initialization methods were used to train the incremental learning GBT model. It should record and count the training error after every model update. Three datasets were trained using random initialization, forgetting feature initialization, median initialization, and information gain-based initialization, respectively. As illustrated in Figure 7, the results showed that no matter which of the proposed built-in tree structure initialization methods were used to train the incremental learning GBT, the model will be updated with iterative training to achieve convergence.

### 3.3. Analysis Results of the Capacity Improvement of the GBT Model

**Figure 8:**
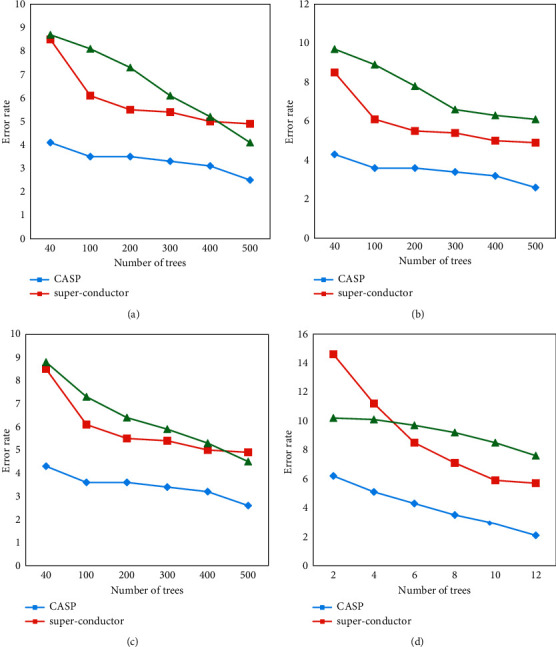
The relationship between the training error of the GBT model and the number of decision trees. (a) Random initialization. (b) Forgetting feature initialization. (c) Median initialization. (d) Information gain-based initialization.

A special feature of the GBT model was that when the user increased the depth of the decision tree or the number of decision trees, the fitting ability of the model would significantly increase. Therefore, this work tested whether GBT models had similar properties. When the depth of the decision tree was fixed to 10 and the number of decision trees was increased, the training error results of the GBT model are shown in Figure 8. The results showed that under the condition that the number of decision trees increased, the fitting ability of the GBT model would be greatly improved.

**Figure 9:**
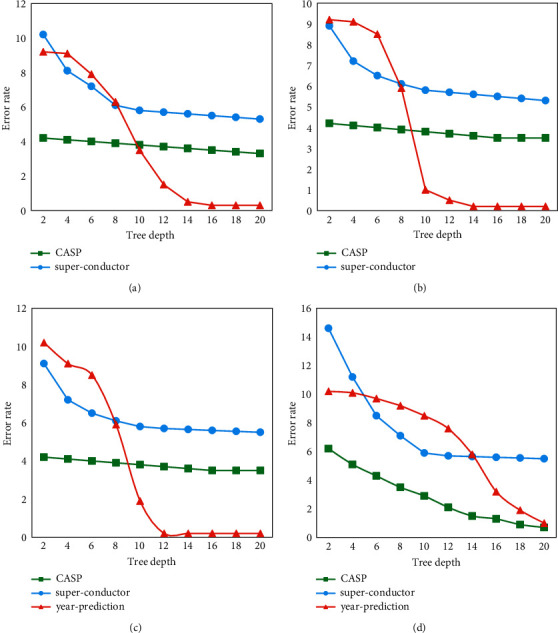
The relationship between the training error of the GBT model and the depth of the decision tree. (a) Random initialization. (b) Forgetting feature initialization. (c) Median initialization. (d) Information gain-based initialization.

Similarly, when the number of decision trees was fixed at 40, the depth of decision trees was gradually increased. The training error results of the GBT model are shown in Figure 9. The results suggested that under the condition that the depth of the decision tree increased, the fitting ability of the GBT model would also be greatly improved.

The above two experiments proved that the GBT model could increase the capacity of the model by increasing the depth or number of trees.

### 3.4. Statistics of Basic Information of Cases

A total of 2,364 patients undergoing CABG were included in this work. The average age of the included patients was (61.21 ± 11.24) years old; the proportion of men was 53.34%, and the proportion of women was 46.65%. There were 18 patients with a history of previous cardiac surgery, accounting for 0.76%; 255 patients with moderate renal impairment, accounting for 10.78%; 1765 patients with grade II cardiac function, accounting for 74.28%; and patients undergoing single coronary artery surgery were 1962 cases, accounting for 82.99%. The basic information of the cases was given in Table 3.

### 3.5. Grouped Based on CABG Risk Prediction System

According to the CABG risk prediction model for stratification, the stratification points selected by the quartile method were 0, 2, and 5, which can be divided into 4 risk stratifications. When the score was ≥6, it was a high-risk stratification (Table 4).

After stratification, it was found that there were 1382 patients with a score <0, 463 patients with a score of 0 ≤ score ≤ 2, 252 patients with a score of 2 < score ≤ 5, and 267 patients with a score >5. The stratified results of the CABG risk prediction model were used for grouping, and the corresponding stratification was four groups: A, B, C, and D. Groups A, B, and C were the low-intermediate risk group, and D was the high-risk group. The grouping results are shown in Table 5. The results showed that there were 25 patients with actual in-hospital deaths, and the in-hospital mortality rate was 1.05%. The mortality rate predicted by the CABG risk prediction system model was 2.67 ± 1.82% (95% CI (2.87, 2.98)), which was higher than the actual value. Among different risk stratification subgroups, the CABG risk prediction system assessment was credible only in group B with AUC = 0.763 > 0.7. In group B, 3 patients actually died, the actual mortality rate was 0.33%, and the model predicted mortality rate was 0.96 ± 0.78 (95% CI (0.82, 0.87)), which overestimated the mortality rate of patients in group B.

In this work, the selected patients were randomly rolled into a modeling group and a validation group according to the ratio of 6 : 4. After the model was established, the calibration and discrimination of the model predictions were verified using the data of the patients in the validation group. According to the CABG risk prediction model, a score ≤1 was differentiated into a low-risk group; a score of 2–5 was differentiated into an intermediate-risk group; and a score ≥6 was differentiated into a high-risk group. The ability of the CABG risk prediction model to predict the mortality risk of these three groups of patients was separately verified. The 95% CIs of the CABG risk prediction system for the low-risk group, intermediate-risk group, and high-risk group were 0.82–0.87, 3.16–3.89, and 8.63–8.98, respectively. This suggests that the CABG risk prediction system has a better ability to predict surgical risk in dangerous subgroups [25–28].

All patients included in this work were risk stratified according to the ESC/EACTS clinical guidelines for myocardial revascularization [29, 30]. The results showed that the CABG risk prediction system overestimated its mortality rate for subgroup. Hung et al. [31] have developed and verified a simple risk score based on clinical variables, which can accurately predict the risk of complications in patients undergoing cardiac surgery before surgery and have a similar predictive function as this study.

Predicting the future development direction of diseases will be based on polymorphic data, that is, structured data such as text, data center, image, ECG center data, time-series data, and unstructured data. It is an important technical challenge to integrate and predict such polymorphic data. With the research results of this project, it will cooperate with many doctors to optimize the model of the risk assessment system and improve the intelligence and accuracy of prediction.

### 3.6. Model Calibration Verification

**Figure 10:**
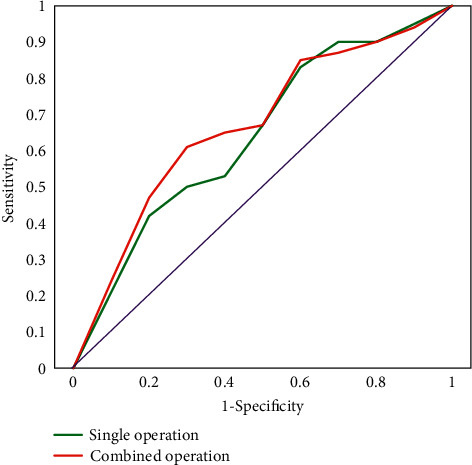
ROC curve of CABG risk prediction model.

The degree of calibration of the model was verified by using the H-L goodness of fit. If the *P* value of H-L >0.05, it indicated that the model had a good degree of calibration. In this work, the overall H-L goodness-of-fit test *P* value was 0.06, which was greater than 0.05, showing that the CABG risk prediction system model fit was good. The *P* values of H-L in different subgroups A, B, and C of the CABG risk prediction system model were 0, 0.47, and 0.025, respectively, all less than 0.05, suggesting that the CABG risk prediction system was poorly calibrated among different subgroups A, B, and C. The *P* value for H-L in group D was 0.153, which was greater than 0.05, indicating that the CABG risk prediction system was well calibrated in group D. In the entire patient cohort, the CABG risk prediction system (AUC = 0.727 > 0.70) had a better discrimination. Among subgroups with different risk levels, the CABG risk prediction system achieved better discrimination only in patients with AUC = 0.763 > 0.7 in class B patients. In the operation type grouping, the AUC values of the CABG risk prediction model in single bypass surgery and bypass combined with other surgery groups were 0.778 and 0.782, respectively, which were all greater than 0.7, indicating that the CABG risk prediction system had the corresponding ability to discriminate in the operation type grouping (Figure 10).

Gunertem et al. [32] collected the perioperative data from 550 CABG patients and used a surgical risk assessment system to predict the incidence of patient death. The results showed that the mortality rate predicted by the system was 2.69%, H-L test *P*=0.612, and AUC = 0.796. Ultimately, 8 patients had in-hospital deaths with a mortality rate of 1.50%. The system was able to predict the center's patient mortality rate well, with similar results to this work.

## 4. Conclusions

In this work, it collected the clinical data of 2364 routine CABG patients in our hospital from January 2019 to August 2021. The AI and big data technologies were adopted to build a CABG risk prediction model, which was adopted to predict the risk of complications in CABG patients. It successfully constructed a CABG risk prediction system that overestimated the mortality rate of intermediate-risk patients. The disadvantage was that it was a single-center retrospective study with a limited number of patients, and a multicenter study was required to include a large number of samples to confirm the conclusions. The risk prediction system of CABG needs to be improved, and the risk prediction system should be continuously developed and innovated to be suitable for different types of heart diseases. In a word, this study included the cases of coronary artery bypass grafting for retrospective study and provided a theoretical basis for risk prediction of perioperative complications.

## Figures and Tables

**Table 1:** Test results of the four models.

	GBT	RF	LR	KNN
Precision	0.87	0.84	0.81	0.73
F1-score	0.89	0.82	0.83	0.78
AUC	0.94	0.85	0.89	0.82

**Table 2:** Evaluation results of GBT model.

	Negatives	Positives	Average (total)
Precision	0.82	0.92	0.85
Recall	0.89	0.84	0.87
F1-score	0.86	0.87	0.86
Amount of data	2143	2876	5019

**Table 3:** Statistics of basic information of cases ($\bar{x}$ ± *s*, *n* (%)).

Item	Data
Age (years old)	61.21 ± 11.24

*Gender*	
Males	1261 (53.34)
Females	1103 (46.65)
History of smoking	856 (36.21)
Diabetes	259 (10.95)
Hypertension	385 (16.28)
History of myocardial infarction	1284 (54.31)
History of previous cardiac surgery	18 (0.76)
Chronic obstructive pulmonary disease	167 (7.06)
Endocarditis	5 (0.21)

*Kidney function*	
Moderate damage	255 (10.78)
Serious injury	836 (35.36)
Normal	1273 (53.84)
Dialysis	

*Ejection fraction*	
Normal (>50%)	1892 (80.03)
Low (<30%)	265 (11.21)
Medium (30–50%)	207 (8.75%)

*Pulmonary pressure*	
Normal (<30 mmHg)	1835 (77.62)
Moderate (30–55 mmHg)	416 (17.59)
Severe (>55 mmHg)	113 (4.78)

*Cardiac function grade*	
I	346 (14.63)
II	1765 (74.28)
III	231 (9.77)
IV	22 (0.93)

*Surgery method*	
Single bypass	1962 (82.99)
Bypass combined with another surgery	335 (14.17)
Bypass combined with more than one other procedure	67 (2.83)
Operation time (h)	4.67 ± 1.32
Extracorporeal circulation time (min)	134.78 ± 35.81
Aortic occlusion time (min)	98.32 ± 21.93
Postoperative drainage volume (mL)	1362.72 ± 353.64
Postoperative tracheal intubation time (h)	29.83 ± 8.76
Length of hospital stay (d)	35.78 ± 12.65

**Table 4:** Stratified results of CABG risk prediction model.

Item	CABG risk prediction model				*P* value
	Score = 0 (*n* = 1382)	0 ≤ score ≤ 2 (*n* = 463)	2 < score ≤ 5 (*n* = 252)	Score >5 (*n* = 267)	
Age (years old)	59.21 ± 13.28	61.25 ± 14.56	63.25 ± 16.24	58.21 ± 10.24	<0.05

Gender					<0.05
Males	654 (47.32)	341 (73.65)	124 (49.20)	142 (53.18)	
Females	728 (52.67)	122 (26.34)	128 (50.79)	125 (46.81)	
History of smoking	452 (32.70)	261 (56.37)	124 (49.20)	19 (7.11)	<0.05
Diabetes	145 (10.49)	56 (12.09)	42 (16.66)	16 (5.99)	<0.05
Hypertension	214 (15.48)	85 (18.35)	46 (18.25)	40 (14.9)	<0.05
History of myocardial infarction	784 (56.72)	271 (58.53)	152 (60.31)	77 (28.83)	<0.05
History of previous cardiac surgery	7 (0.50)	4 (0.86)	5 (1.98)	2 (0.74)	<0.05
Chronic obstructive pulmonary disease	72 (5.20)	35 (7.55)	27 (10.71)	33 (12.35)	<0.05
Endocarditis	0 (0.00)	0 (0.00)	3 (1.19)	2 (0.74)	<0.05

Kidney function					<0.05
Moderate damage	134 (9.69)	75 (16.19)	28 (11.11)	18 (6.74)	
Serious injury	453 (32.77)	274 (59.17)	56 (22.22)	53 (19.85)	
Normal	686 (49.63)	372 (80.34)	134 (53.17)	81 (30.33)	
Dialysis					

Ejection fraction					<0.05
Normal (>50%)	1236 (89.43)	352 (76.02)	154 (61.11)	150 (56.17)	
Low (<30%)	78 (5.6)	56 (12.09)	72 (28.57)	75 (28.08)	
Medium (30–50%)	68 (4.92)	55 (11.87)	26 (10.31)	42 (15.73)	

Pulmonary pressure					<0.05
Normal (<30 mmHg)	1076 (77.85)	326 (70.41)	215 (85.31)	218 (81.64)	
Moderate (30–55 mmHg)	159 (11.50)	58 (12.52)	18 (7.14)	31 (11.61)	
Severe (>55 mmHg)	147 (10.63)	79 (17.06)	17 (6.74)	18 (6.74)	

Cardiac function grade					<0.05
I	78 (5.64)	246 (53.13)	135 (53.57)	101 (37.82)	
II	1187 (85.89)	125 (26.99)	74 (29.36)	53 (19.85)	
III	59 (4.26)	53 (11.44)	19 (7.53)	67 (25.09)	
IV	58 (4.19)	39 (8.42)	24 (9.52)	46 (17.22)	

Surgery method					<0.05
Single bypass	1026 (74.24)	412 (88.98)	146 (57.93)	156 (58.42)	
Bypass combined with another surgery	216 (15.62)	27 (5.83)	58 (23.01)	78 (29.21)	
Bypass combined with more than one other procedure	140 (10.13)	24 (5.18)	48 (19.04)	33 (12.35)	
Operation time (h)	4.17 ± 1.35	5.47 ± 1.33	4.27 ± 1.37	6.67 ± 1.38	<0.05
Extracorporeal circulation time (min)	152.74 ± 33.81	171.75 ± 35.21	125.75 ± 35.81	160.78 ± 34.83	<0.05
Aortic occlusion time (min)	98.32 ± 21.96	91.32 ± 23.93	92.32 ± 24.93	99.32 ± 23.94	<0.05
Postoperative drainage volume (mL)	1458.72 ± 363.65	1565.76 ± 356.65	1368.74 ± 363.63	1652.73 ± 363.63	<0.05
Postoperative tracheal intubation time (h)	31.83 ± 8.76	25.83 ± 8.78	22.83 ± 8.76	25.93 ± 8.76	<0.05
Length of hospital stay (d)	37.78 ± 12.15	34.78 ± 12.45	39.78 ± 12.35	31.78 ± 12.67	<0.05

**Table 5:** Actual mortality rate and mortality rate predicted by CABG risk prediction model.

Group	Total number	Number of deaths	Actual mortality rate	Predicted mortality rate	Predicted mortality rate (95% CI)	AUC	H-L test (*χ*^2^)	H-L test (*P* value)
A	1516	0	0	0	0	0	0	0
B	563	3	0.33	0.96 ± 0.78	(0.82, 0.87)	0.521	0.753	0.34
C	368	5	0.20	3.27 ± 0.83	(3.16, 3.89)	0.763	4.621	0.032
D	187	17	0.29	8.78 ± 2.19	(8.63, 8.98)	0.362	8.373	0.165
Total	2364	25	0.68	2.67 ± 1.82	(2.87, 2.98)	0.732		

## Data Availability

All data included in this study are available upon request by contact with the corresponding author.
